# Case report: Aberrant fecal microbiota composition of an infant diagnosed with prolonged intestinal botulism

**DOI:** 10.1186/s13099-024-00614-y

**Published:** 2024-04-05

**Authors:** François P. Douillard, Yağmur Derman, Ching Jian, Katri Korpela, Harri Saxén, Anne Salonen, Willem M. de Vos, Hannu Korkeala, Miia Lindström

**Affiliations:** 1 Department of Food Hygiene and Environmental Health, Faculty of Veterinary Medicine, University of Helsinki, Helsinki, Finland; 2 Human Microbiome Research Program, Faculty of Medicine, University of Helsinki, Helsinki, Finland; 3 New Children’s Hospital, Pediatric Research Center, University of Helsinki and Helsinki University Hospital, Helsinki, Finland; 4 Laboratory of Microbiology, Wageningen University & Research, Wageningen, The Netherlands

**Keywords:** Infant botulism, *Clostridium botulinum*, Botulinum neurotoxin, Fecal microbiota, 16S rRNA gene sequencing

## Abstract

**Background:**

Intestinal botulism is primarily reported in small babies as a condition known as infant botulism. The condition results from the ingestion of environmental or foodborne spores of botulinum neurotoxin (BoNT) producing *Clostridia*, usually *Clostridium botulinum*, and subsequent spore germination into active botulinum neurotoxinogenic cultures in the gut. It is generally considered that small babies are susceptible to *C. botulinum* colonization because of their immature gut microbiota. Yet, it is poorly understood which host factors contribute to the clinical outcome of intestinal botulism. We previously reported a case of infant botulism where the infant recovered clinically in six weeks but continued to secrete *C. botulinum* cells and/or BoNT in the feces for seven months.

**Case presentation:**

To further understand the microbial ecology behind this exceptionally long-lasting botulinum neurotoxinogenic colonization, we characterized the infant fecal microbiota using 16S rRNA gene amplicon sequencing over the course of disease and recovery. *C. botulinum* could be detected in the infant fecal samples at low levels through the acute phase of the disease and three months after recovery. Overall, we observed a temporal delay in the maturation of the infant fecal microbiota associated with a persistently high-level bifidobacterial population and a low level of *Lachnospiraceae*, *Bacteroidaceae* and *Ruminococcaceae* compared to healthy infants over time.

**Conclusion:**

This study brings novel insights into the infant fecal composition associated with intestinal botulism and provides a basis for a more systematic analysis of the gut microbiota of infants diagnosed with botulism. A better understanding of the gut microbial ecology associated with infant botulism may support the development of prophylactic strategies against this life-threatening disease in small babies.

**Supplementary Information:**

The online version contains supplementary material available at 10.1186/s13099-024-00614-y.

## Background

The human gut microbiota (HGM) consists of an astonishingly large number of phylogenetically diverse bacterial species. The healthy HGM has been extensively investigated notably by large research consortia across the globe, such as the Human Intestinal Tract (MetaHIT) [1] and the US Human Microbiome Project (HMP) [2]. Besides its core function that is to assimilate nutrients, such as plant carbohydrates [3] and glycans [4], the HGM plays other relevant biological functions for the human host, including bile acid metabolism [5], biosynthesis of short-chain fatty acids (SFCA) [6], biosynthesis of vitamins [5], immunomodulation [7, 8], and protection against pathogens [9, 10]. The HGM is constantly shaped by environmental or other external factors [11, 12] and host factors [13] and it changes through the different stages of life [14–17]. Deleterious changes in the HGM composition have been associated with some gastrointestinal disorders and diseases [18, 19] as well as systemic diseases [20], illustrating the intricate link between human health and HGM. This has led to major research efforts in developing biotherapeutic agents, such as prebiotics and health-promoting bacteria (probiotics), to re-shape dysbiotic HGM [21–24].

The infant HGM is unstable and has low microbial richness and diversity compared to the adult HGM [15, 16, 25]. In healthy adults, the gut is typically and predominantly colonized by two microbial phyla: Bacteroidetes and Firmicutes [26], whereas the infant gut is initially more abundant in Actinobacteria [27]. Underneath this over-simplified snapshot lie major temporal changes and events in microbial colonization, diversity, and dynamics in the infant gut [15, 16]. Microbial colonization and composition of the infant gut are influenced by multiple external factors, such as birth delivery mode [28, 29], antibiotic treatments [30, 31] or diet [32].

Infant botulism results from ingestion of spores of neurotoxinogenic *Clostridia* and subsequent spore germination into active neurotoxinogenic cultures in the gut [33, 34]. It is generally considered that small infants are susceptible to colonization because their immature gut microbiota are unable to outcompete neurotoxinogenic *Clostridia*, such as *Clostridium botulinum*, *Clostridium butyricum* and *Clostridium baratii*. In addition, other factors like bile acids or probiotic micro-organisms may impact spore germination, growth, toxin production or toxin potency of neurotoxinogenic *Clostridia* [35, 36]. Typically, most infant botulism cases are diagnosed in infants of less than 6 months of age [37]. Upon colonization of, presumably, the lumen of the large intestine by BoNT-producing *Clostridia* [38, 39], BoNT is produced in situ and intoxicates the host, resulting in flaccid paralysis known as botulism [40]. In adults, a similar condition (toxicoinfectious botulism) may be preceded by intestinal surgery, intestinal disorders, or antimicrobial treatment [41–43], all assumed to re-shape the gut microbiome and to provide a competitive edge for *C. botulinum* in a manner analogous to *Clostridioides difficile* infection. Clinical presentation, diagnosis, treatment, and prognosis of infant botulism have been well documented over the years [40, 44]. There are a number of well-established etiological factors that increase the risk of developing infant botulism, including diet (consumption of honey that may contain clostridial spores) [45, 46], constipation [47], environment (excess of dust particles) [45, 48] and geographical location (environmental spore load) [33, 47, 49].

Laboratory confirmation of infant botulism typically relies on detection of BoNT and/or isolation of neurotoxinogenic *Clostridia* from stool samples [44, 50]. The development of next-generation sequencing technologies has been instrumental in analyzing microbial composition of stool samples without the need to isolate and cultivate gut microbes. It provided significant insights into the association of the HGM with a number of diseases and disorders, such as metabolic disorders [51, 52], intestinal diseases [53, 54], and cancer [55, 56]. There is, however, scarce information on the role or impact of the HGM in the context of infant botulism. A single piece of work reported, among others, a lower relative abundance ratio of Firmicutes/Proteobacteria, and a higher relative abundance of *Enterobacteriaceae* in the fecal microbiota of infants diagnosed with botulism compared to the fecal microbiota of healthy infants [57]. There are no studies where the fecal microbiota of infants with botulism were followed over time to define the gut microbial context allowing *C. botulinum* to colonize, transiently persist, and be cleared from the infant gut. Understanding the relationship and dynamics between *C. botulinum* and gut microbiota gives novel insight into the ecology and epidemiology of infant botulism and provides novel perspectives to its prevention and treatment.

We earlier reported a botulism case of 3-month old infant and showed that, despite prompt clinical recovery, *C. botulinum* (Group I) type A persisted and produced BoNT in the infant gut for 27.7 weeks [58]. In contrast, in most infant botulism cases caused by *C. botulinum* type A, the median excretion of *C. botulinum* and the toxin was reported to be 5.9 weeks [59]. To further understand the exceptionally long colonization of the infant gut by *C. botulinum* in this particular case, we performed an extensive genomic analysis of *C. botulinum* isolates collected from the infant feces over time and identified possible pheno-genotypic adaptation traits of *C. botulinum* to the gut environment [60]. In the present work, we complemented our understanding of this infant botulism case by looking at temporal changes in the microbial signatures of the infant gut microbiota composition during the persistence and the clearance of *C. botulinum* from the infant gut. Based on well-established phylogenetic analytical tools, we observed a delay in the maturation of the infant fecal microbiota over time associated with a persistently high-level bifidobacterial population, and identified bacterial species possibly linked with the clearance of *C. botulinum* from the infant gut. To our knowledge, this is the first report where the gut microbiota composition of an infant diagnosed with infant botulism is examined temporally throughout various stages of the disease. It brings novel insights into the microbial ecological factors that may trigger intestinal botulism. While this case report depicts a single case, thus individual deviations in microbiota composition in other patients are likely, our report suggests that systematic large-scale approaches may help to identify patterns within the gut microbiota composition of infants associated with intestinal botulism, possibly leading to the development of preventive and therapeutic measures.

## Materials and methods

### Fecal samples

**Fig. 1:**
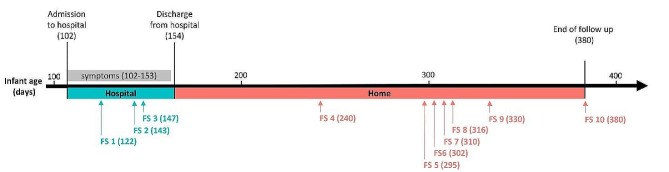
Overview of the fecal sampling related to the presented infant botulism case. Only the first three fecal samples were collected at the hospital. Subsequent samples were collected at home by the parents. The infant age is indicated in brackets. FS, fecal sample

We analyzed 10 fecal samples collected over a period of 259 days from a case of infant botulism in Finland [58]. The infant was admitted to hospital at the age of 102 days and discharged at the age of 154 days. The first stool sample (infant age 122 days) related to a clinical stage when the infant displayed severe symptoms of botulism, whereas the very last sample (infant age 380 days) related to a stage where the infant had clinically fully recovered, had been discharged from hospital for more than 200 days, and tested negative for the presence of *C. botulinum* vegetative cells and spores and negative for BoNT in the feces [58]. All fecal samples analyzed in this work were collected after the infant had received both antibiotic treatment (ceftriaxone at infant age of 103–106 days) and antiviral medication (acyclovir at 102–108 days) (Fig. 1). The first three samples (infant age 122, 143 and 147 days) were collected at the hospital. All subsequent samples were collected at home, picked up and transported to the laboratory by a member of the lab and then stored in freezers. Informed consent was obtained from the patient’s parents.

### DNA extraction, library preparation and sequencing

Fecal bacterial genomic DNA was extracted using the Quick-DNA Fecal/Soil Microbe Kits (Zymo Research, CA, USA) as per manufacturer’s instructions. DNA was quantified using NanoDrop™ 2000 Spectrophotometer (ThermoFischer Scientific, MA, USA) and stored at -20 °C. The hypervariable V3-V4 regions of the 16S rRNA gene were amplified using primers 341 F/758R and were further processed for library preparation using a modified protocol by Illumina and sequenced by Illumina HiSeq 2500 sequencer using HiSeq Rapid SBS Kit v2 (2 × 250 bases), as previously described [61–63]. Paired-end read sequencing using the Illumina MiSeq platform was performed at the Institute for Molecular Medicine Finland (FIMM, University of Helsinki, Finland).

### Bioinformatics and statistical data analysis

Demultiplexed reads after adaptor removal by cutadapt [64] were processed using DADA2 [65], where *truncLenF* and *truncLenR* were set to 270 and 230, respectively, and reads with a number of expected errors higher than two were discarded. The forward and reversed reads were subsequently merged with a minimum overlap of 25 nucleotides to generate amplicon sequence variants (ASVs). Taxonomy was assigned to all ASVs using a pre-trained naïve Bayes classifier implemented in DADA2 (*assignTaxonomy* function with default settings) against the SILVA 138 reference database [66]. Species assignment was performed using DADA2 by exact string matching (*addSpecies* function with the argument “allowMultiple = FALSE”) against the SILVA v138.1 species assignment training database [67]. Of note, the ASV belonging to *Clostridium botulinum* was annotated as *Clostridium sensu stricto* 18 using the SILVA 138 database (Table S2), as confirmed by NCBI BLAST [68]. Principal coordinate analysis (PCoA) plot based on the Bray-Curtis dissimilarity was employed to visualize the differences in overall microbiota composition (β-diversity) between sampling points. Statistical significance of the difference in microbiota β-diversity between the hospital and home phases was tested using permutational multivariate analysis of variance (PERMANOVA; *adonis*2 function in the *vegan* package [69] with 999 permutations based on the Bray-Curtis dissimilarity). Microbiota α-diversity (observed richness, Shannon and inverse Simpson diversity indices) was estimated using the *vegan* package [69]. Statistical significance of the difference in microbiota α-diversity between the hospital and home phases was tested by calculating Tau-*U*, a non-overlap index designed for analysis of single-case research data [70]. *P*-values < 0.05 were considered significant for the analyses of microbiota α- and β-diversity. Given the single-case nature of this study compounded by the volatility of individual microbial taxa, we opted for visual and/or descriptive analysis for changes in specific microbial taxa over time to provide high-granularity information.

## Results and discussion

### Fecal microbiota composition during the course of infant botulism

**Fig. 2:**
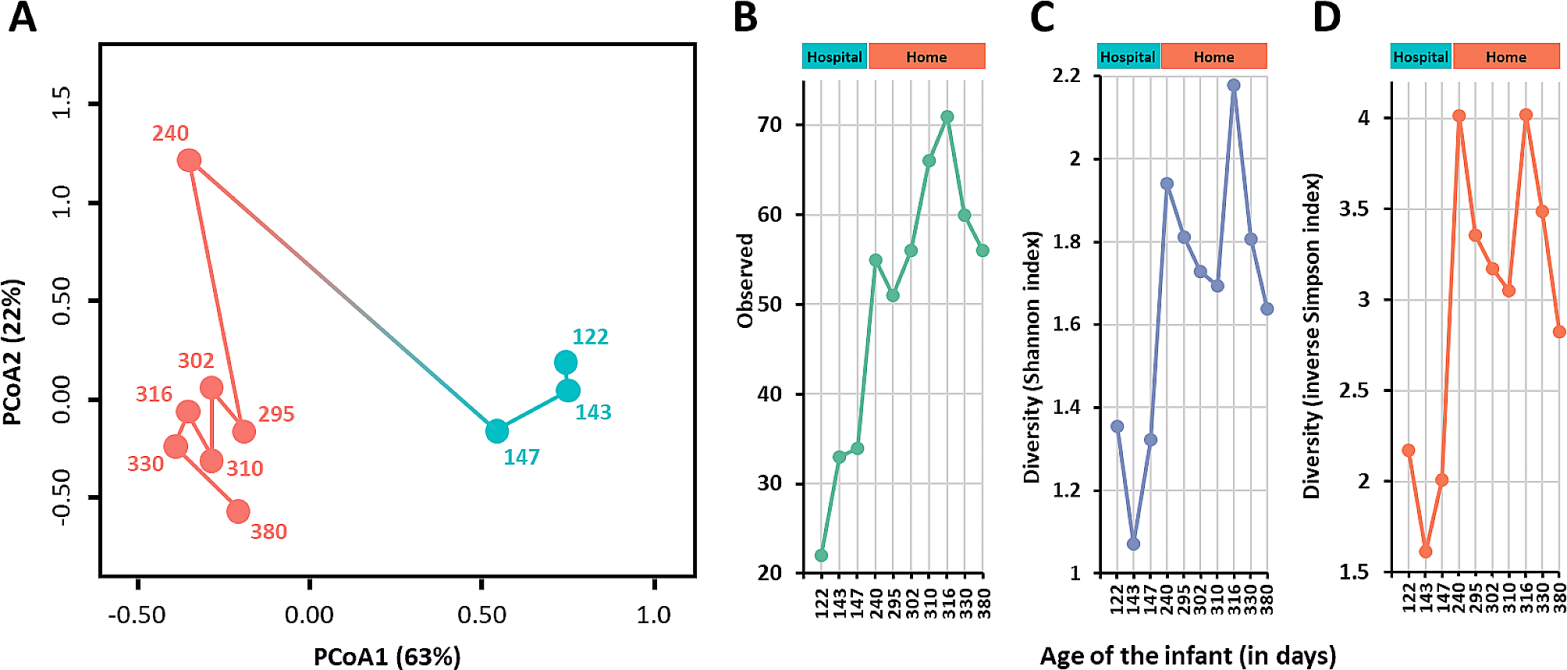
Overview of the fecal microbiota composition of the infant stool samples over time. (**A**) Principal coordinate analysis (PCoA) plot of microbiota variation based on the Bray-Curtis dissimilarity matrix. Blue, samples collected at hospital; red, samples collected in the infant’s home. For each sample, the infant age (in days) was indicated as numbers. Sample collection phase (hospital vs. home) explained 61% of the microbiota variation (PERMANOVA *p* = 0.006). (**B**.**-D**) Microbiota α-diversity of the samples was significantly lower during hospitalization (all Tau-*U* = 1, *p* = 0.017) according to observed richness (**B**), Shannon diversity index (**C**), and inverse Simpson diversity index (**D**)

We analyzed the microbial composition of 10 stool samples collected from an infant botulism case [58] over a period of 7 months, covering different stages of the disease. The metrics and statistics related to the 16S rRNA gene amplicon sequencing of the 10 samples are presented in Table S1. Additional metadata related to the fecal samples are available in our previous work [58]. The overall microbiota composition and within-sample diversity fluctuated over time, indicating that major changes occurred in the infant fecal microbiota composition during and after the course of the disease (Fig. 2). The overall microbiota compositions were similar in the fecal samples collected when the infant was symptomatic and treated at the hospital (102–154 days), as reflected visually in the PCoA plot showing clustering of the first three samples (PERMANOVA *p* = 0.006, 61% microbiota variation explained by hospital versus home; Fig. 2A). Microbiota α-diversity (observed richness, the Shannon and inverse Simpson diversity indices) was significantly lower during the hospital phase compared to the home phase (all Tau-*U* = 1, *p* = 0.017; Fig. 2B-D). This may be explained at least partly by the early antibiotic treatments, ceased oral food intake, and/or a more controlled environmental microbial load during the hospital phase compared to the home phase. In the absence of samples taken prior to the hospital stay, it remains unclear to what extent these factors impacted the infant gut microbiota. Interestingly, fecal sample 2 (infant age 143 days) appeared to be the “tipping point” in terms of diversity indices (lowest Shannon diversity index and inverse Simpson diversity index). Such tipping points have been described in adult microbiota in reflecting critical transitions with profound health implications [71].

**Fig. 3:**
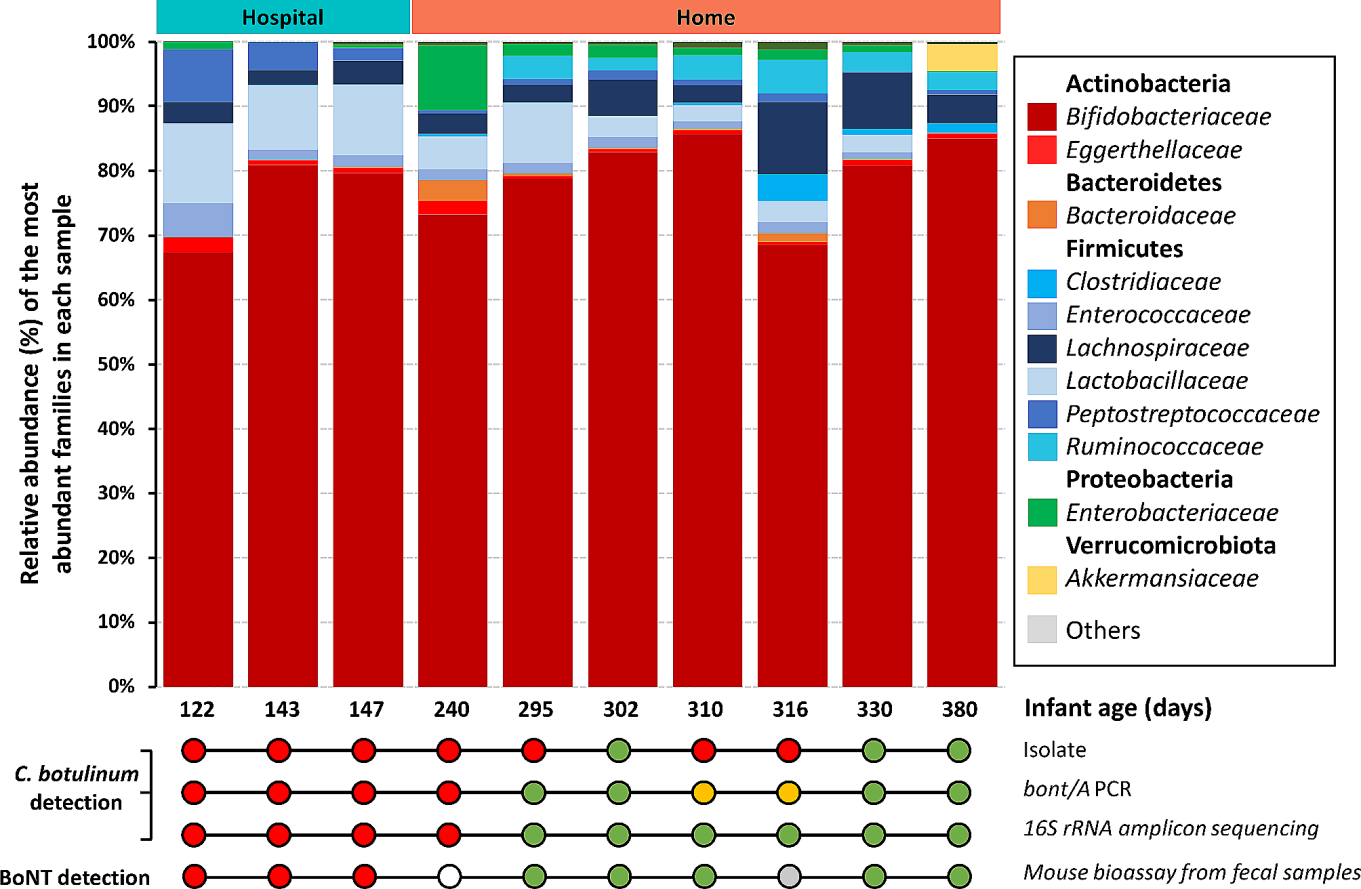
Family-level microbial composition of the infant fecal samples over time. All families shown here were detected at all times, except *Bacteroidaceae*, *Veillonellaceae* and *Akkermansiaceae*. Detection of *C. botulinum* by 16S rRNA gene amplicon sequencing is further detailed in Table 1. All samples were collected after antiviral and antibiotic treatment (administered at infant age 103–108 days). The lower part of the figure shows information related to the detection of *C. botulinum* in the fecal samples based on direct isolation of *C. botulinum*, PCR detection of *bont/A* and BoNT detection by mouse bioassay in the different samples and were published earlier [58]. Green dot, negative; orange dot, inconclusive; red dot, positive; white dot, not tested; grey dot, unspecific symptoms

The phylum-level composition of the infant fecal microbiota (Fig. 3) revealed that the phylum with the highest abundance across all samples was Actinobacteria, consisting of *Bifidobacteriaceae* and *Eggerthellaceae*. At the order level, *Bacteroidales*, *Bifidobacteriales*, *Clostridiales*, *Coriobacteriales*, *Enterobacteriales*, *Lachnospirales*, *Lactobacillales*, *Oscillospirales* and *Peptostreptococcales* were detected at all times in the infant gut, whereas other orders were intermittently present. We looked at the relative abundance of different families over time in the infant feces and compared to healthy infants with a normal gut microbiota development [15, 72, 73]. While the average relative abundance of *Bifidobacteriaceae* in healthy infants usually decreased to 10–20% by the end of the first year in life [72, 73], *Bifidobacteriaceae* remained at a high level over the age of one year in the infant botulism patient described (86.4%, infant age 380 days) (Fig. 3 and S1).

The relative abundance of *Lactobacillaceae* steadily decreased during the first year of life in our infant (Figure S1), as observed also in healthy infants [15, 73]. During the same period of time in our patient, however, *Bacteroidaceae* remained at low levels in all fecal samples (at most 3.21%), while in the healthy infant gut *Bacteroidaceae* established a larger population over time (an average of 15% of *Bacteroidaceae* in a Finnish infant cohort over the first year) [74]. In the present case, *Lachnospiraceae* and *Ruminococcaceae* marginally increased over time (Figure S1). Typically, *Bacteroidaceae*, *Lachnospiraceae*, and *Ruminococcaceae* outnumbered *Bifidobacteriaceae* by the age of 1 year in healthy infants [15, 73], which clearly contrasts with the fecal microbiota of the infant analyzed in our study. While levels of bifidobacteria were generally seen to decline over the first year of life in many studies, a recent large multicenter study suggested wide variation with some infants showing bifidobacteria-dominant enterotypes for longer periods, illustrating that there is a wide individual diversity [75]. Of note, *Verrucomicrobiales* that include *Akkermansia* sp. showed their highest levels (3.76%) in the last fecal sample collected from our infant, recovered from botulism by the sampling time. The genus *Akkermansia* serves as one of the biomarkers indicating a healthy gut condition (healthy mucus layer) [76]. Whether the emergence of *Akkermansia* sp. promoted clearance of *C. botulinum* from the infant gut, or developed as a consequence, remains to be understood. Overall, the data suggest that the maturation and development of the fecal microbiota was largely delayed in our patient with infant botulism, with a persistently high level of *Bifidobacteriaceae* over time in relation to healthy infants.

### *Clostridium botulinum* persists at low levels in feces in the described case of infant botulism

Depending on the method used for the detection of *C. botulinum* in the infant feces, i.e. detection of *bont/A* by PCR, direct isolation of *C. botulinum* [58], or 16S rRNA gene amplicon sequencing (this study), 4 to 7 fecal samples were positive for *C. botulinum* (Fig. 3). Solely based on 16S rRNA gene amplicon sequencing (this work), the relative abundance of *C. botulinum* in the infant fecal microbiota was at its highest 0.023% in fecal sample 2 (infant age 143 days) and decreased over time, until being under the detection level (Table 1). This is in line with a previous study showing the relative abundance of *C. botulinum* in the feces of infants diagnosed with botulism to be < 0.001 to 0.01% [57]. These values indicate that neurotoxinogenic *C. botulinum* represents, at most, a marginal fraction of the gut microbiota in confirmed botulism cases. Yet, this appears sufficient for host intoxication and systemic paralysis, obviously due to the extremely high potency of BoNT. This illustrates how a very-low-abundant organism present in the gut can still have a consequent impact on the host health, so merely focusing on the most abundant taxa may be insufficient.

**Table 1:** The relative abundance (%) of *C. botulinum* 16S rRNA gene amplicon sequencing reads in the infant fecal samples over time

Sample #	Infant age (days)	*C. botulinum* 16S rRNA
1	122	0.018%
2	143	0.023%
3	147	0.020%
4	240	0.018%
5	295	0.000%
6	302	0.000%
7	310	0.000%
8	316	0.000%
9	330	0.000%
10	380	0.000%

As reported earlier [58], from the age of 154 to 245 days, the infant continued to excrete both neurotoxinogenic *C. botulinum* and BoNT, while not displaying clinical symptoms. This suggests that the infant may have developed mucosal immunity against BoNT over time, similarly to mucosal vaccines shown to prevent mucosal BoNT intoxication [77, 78]. From the age of 295 to 316 days, the clinically fully recovered infant may have excreted non-toxinogenic *C. botulinum* based on *C. botulinum* detection, isolation, and toxicity analysis by the mouse bioassay [58]. In addition to possible mucosal immunity to BoNT, the absence of clinical symptoms in this period of time could be explained by the pheno-genotype of the *C. botulinum* population evolving over time. Indeed, whole-genome sequencing of late *C. botulinum* stool isolates of the current case revealed the presence of multiple mutations in genes coding for the *agr-2* quorum sensing system [60]. The *agr-2* signaling system modulates neurotoxin production in *C. botulinum* strain ATCC 3502 in vitro [79], thus it is possible that a *C. botulinum* population with an impaired *agr-2* quorum-sensing system remained in the infant gut and did not produce BoNT in the gut conditions. Interestingly, we also detected the presence of *C. difficile* in all fecal samples (up to 6.58%). Co-occurrence of the two species has been previously reported in other intestinal botulism cases [80–82]. It remains unclear if the higher relative abundance of *C. difficile* during the hospital phase than at home phase was due to nosocomial infection or contributed to the onset or course of infant botulism.

The low abundance of *C. botulinum* in botulism-confirmed samples may introduce a diagnostic challenge for 16S rRNA amplicon sequencing due to borderline detection sensitivity when investigating infant (intestinal) botulism cases. This warrants a dual approach where DNA-based detection of *C. botulinum* from fecal DNA samples could be conducted by using both 16S RNA amplicon sequencing (comprehensive analysis of the fecal microbiota composition) and real-time PCR (*C. botulinum* detection and diagnosis) in parallel (Fig. 3). Likely, the timing of fecal sampling is also critical, and *C. botulinum* appears to be more likely detected by 16S rRNA gene amplicon sequencing upon disease onset and at the time when symptoms are the most prominent. For longitudinal studies, therefore, multiple detection techniques should be preferred.

### Bifidobacterial population structurally changed over time but remained at high level

**Fig. 4:**
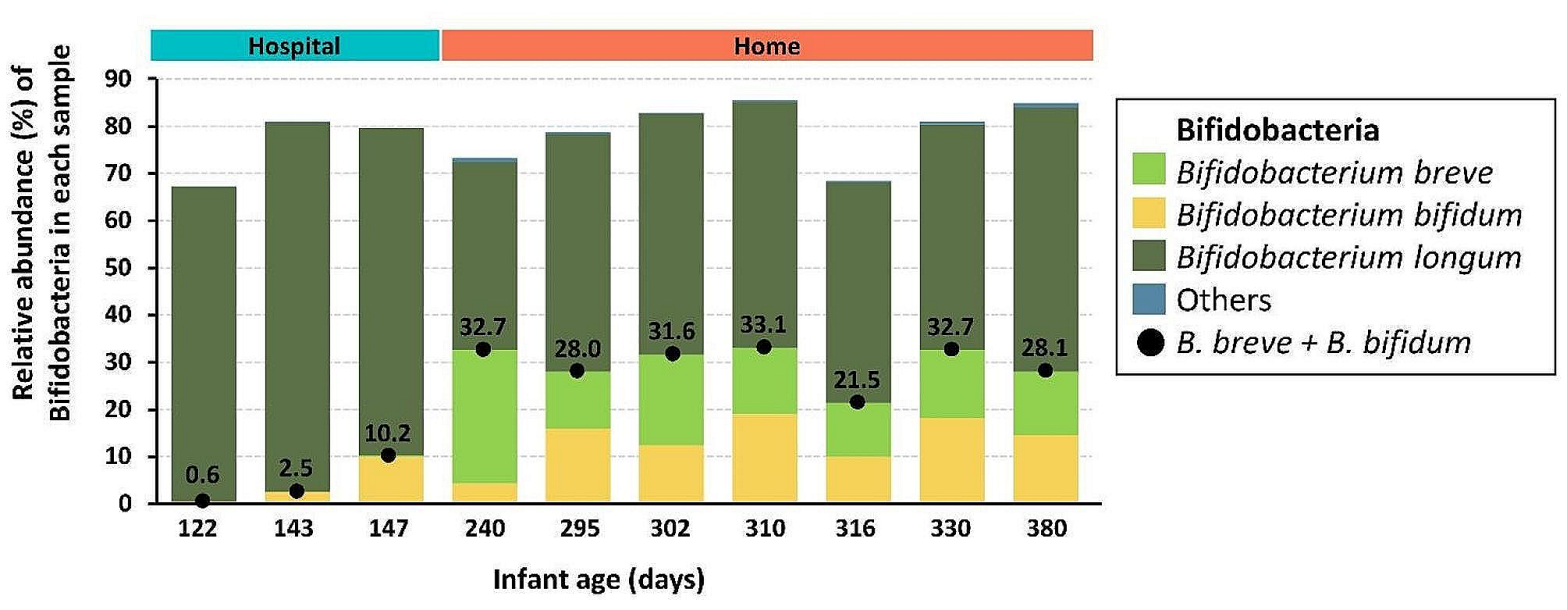
Relative abundance of the bifidobacteria population in the infant fecal samples over time. The cumulated relative abundance of *B. breve* and *B. bifidum* for each sample is indicated above the plot

The healthy adult gut is typically and predominantly colonized by the microbial phyla Bacteroidetes and Firmicutes [26], whereas the infant gut is more abundant with Actinobacteria [27]. Within Actinobacteria, *Bifidobacteria* have been shown to colonize and persist in the infant gut microbiota [83] and to play an important role in the development and maturation of the gut microbiota. They also have a protective role in the intestinal barrier function and contribute to immuno-modulation [84]. *Bifidobacterium longum*, *Bifidobacterium breve*, and *Bifidobacterium bifidum* are among the most prevalent species in the infant gut [27, 85, 86]. To understand their possible roles in infant botulism, we particularly examined the bifidobacterial populations present in the infant feces over time (Fig. 4). Overall, the bifidobacterial population of the infant gut remained at high levels for a longer time than observed in healthy infants [15]. Specifically, during the first year of life, the diet change from milk to solid and diverse food typically prompts a decline in *Bifidobacteriaceae* and *Lactobacillaceae* and an increase in *Lachnospiraceae*, *Bacteroidaceae* and *Ruminococcaceae* [15]. As indicated above, the persistence of a high bifidobacterial population in the infant gut suggests a delayed maturation of the infant fecal microbiota. The possible role of delayed maturation of the gut microbiota in the development, course, and recovery of infant botulism, and the relevance of early antibiotic treatments therein, warrants further investigation. On the same line, it remains unclear whether *C. botulinum*, BoNT, or host factors may have impacted the gut microbiota over time.

In terms of population structure, the bifidobacterial population in the diseased infant consisted of only few bacterial species as described for healthy infants [27]. While one bifidobacterial species dominated this population in the early fecal samples (Fig. 4), others such as *B. breve* and *B. bifidum* colonized the infant gut at a later stage in parallel with gradual clearance of toxinogenic colonization (Fig. 4). With existing data it is unclear if the colonization of the gut by *B. breve* and *B. bifidum* contributed to clearance of infant botulism, or if clearance of the toxinogenic colonization allowed *B. breve* and *B. bifidum* to thrive, and if and how other host or environmental factors may be involved. Since some strains of *Bifidobacteria* have been shown to inhibit the growth of *C. botulinum in vitro* [87], it is tempting to speculate that some *Bifidobacteria* species or strains may confer a protective effect against infant botulism by preventing the germination of *C. botulinum* spores and their colonization of the gut. Animal model-based assays will provide further evidence to support this hypothesis.

## Concluding remarks

Here we aimed at further understanding of factors underlying an extremely long-term botulinum neurotoxinogenic colonization and different stages of infant botulism by looking at the infant gut microbiota composition over the course of the disease. We observed a delayed maturation of the infant gut microbiota with a persistently high bifidobacterial population and a low level of *Lachnospiraceae*, *Bacteroidaceae* and *Ruminococcaceae* over time compared to healthy infants where *Bifidobacteriaceae* usually decreased to a relative abundance of 10–20%, and *Bacteroidaceae*, *Lachnospiraceae*, and *Ruminococcaceae* outnumbered *Bifidobacteriaceae* by the age of one year. We suggest that the delay in the maturation of the infant gut microbiota may have explained the exceptionally long colonization and excretion of *C. botulinum* and BoNT in the infant gut. The relative abundance and population structure of *Bifidobacteria* is likely to play a central role during the course of toxinogenic colonization in infant botulism. *B. breve* and *B. bifidum* appeared to be temporally associated in the clearance of *C. botulinum*, highlighting the protective role of some bifidobacterial species against pathogens. Yet, it remains to be elucidated if and how *B. breve* and *B. bifidum* may interact with *C. botulinum* and if host or environmental factors are concomitantly involved. To conclude, this work provides valuable insights into the microbiota changes occurring during and after *C. botulinum* colonization in the infant gut. It is likely that other infant fecal microbiota composition signatures are also associated with infant botulism. Therefore, we advocate a more systematic analysis of the gut microbiota of infants diagnosed with botulism in an effort to further identify recurrent bacterial signatures associated with botulism and to develop prophylactic strategies and measures to prevent this severe disease in small children.

### Electronic supplementary material

Below is the link to the electronic supplementary material.

**Supplementary Figure S1**: Relative abundance of relevant bacterial families over time

**Supplementary Table S1**: 16S rRNA gene sequencing statistics of all fecal samples analyzed in the present study. Only the first three sample were collected when the infant was at the hospital

**Supplementary Table S2**: Taxonomic table containing the data obtained after processing the sequencing reads

## Data Availability

The dataset generated during the current study will be available in NCBI databases under the BioProject accession number PRJNA921941.

## Abbreviations

BoNT: Botulinum neurotoxin
HGM: Human gut microbiota
PCR: Polymerase chain reaction

## References

[1] Qin J, Li R, Raes J, Arumugam M, Burgdorf KS, Manichanh C (2010). A human gut microbial gene catalogue established by metagenomic sequencing. Nature.

[2] Consortium THMP (2012). Structure, function and diversity of the healthy human microbiome. Nature.

[3] Flint HJ, Scott KP, Duncan SH, Louis P, Forano E (2012). Microbial degradation of complex carbohydrates in the gut. Gut Microbes.

[4] Koropatkin NM, Cameron EA, Martens EC (2012). How glycan metabolism shapes the human gut microbiota. Nat Rev Microbiol.

[5] Rowland I, Gibson G, Heinken A, Scott K, Swann J, Thiele I (2018). Gut microbiota functions: metabolism of nutrients and other food components. Eur J Nutr.

[6] Silva YP, Bernardi A, Frozza RL. The role of short-chain fatty acids from gut microbiota in gut-brain communication. Front Endocrinol. 2020;11. 10.3389/fendo.2020.00025 10.3389/fendo.2020.00025 PMC7005631 32082260

[7] Kaplan JL, Shi HN, Walker WA (2011). The role of microbes in developmental immunologic programming. Pediatr Res.

[8] Zheng D, Liwinski T, Elinav E (2020). Interaction between Microbiota and immunity in health and disease. Cell Res.

[9] Panwar RB, Sequeira RP, Clarke TB (2021). Microbiota-mediated protection against antibiotic-resistant pathogens. Genes Immun.

[10] Ubeda C, Djukovic A, Isaac S (2017). Roles of the intestinal microbiota in pathogen protection. Clin Transl Immunol.

[11] Gacesa R, Kurilshikov A, Vich Vila A, Sinha T, Klaassen MAY, Bolte LA (2022). Environmental factors shaping the gut microbiome in a Dutch population. Nature.

[12] Ahn J, Hayes RB (2021). Environmental influences on the human microbiome and implications for noncommunicable disease. Annu Rev Public Health.

[13] Kurilshikov A, Medina-Gomez C, Bacigalupe R, Radjabzadeh D, Wang J, Demirkan A (2021). Large-scale association analyses identify host factors influencing human gut microbiome composition. Nat Genet.

[14] Odamaki T, Kato K, Sugahara H, Hashikura N, Takahashi S, Xiao J-z (2016). Age-related changes in gut microbiota composition from newborn to centenarian: a cross-sectional study. BMC Microbiol.

[15] Derrien M, Alvarez A-S, de Vos WM (2019). The gut microbiota in the first decade of life. Trends Microbiol.

[16] Rodríguez JM, Murphy K, Stanton C, Ross RP, Kober OI, Juge N (2015). The composition of the gut microbiota throughout life, with an emphasis on early life. Microb Ecol Health Dis.

[17] Jeffery IB, Lynch DB, O’Toole PW (2016). Composition and temporal stability of the gut microbiota in older persons. ISME J.

[18] de Vos WM, de Vos EA (2012). Role of the intestinal microbiome in health and disease: from correlation to causation. Nutr Rev.

[19] de Vos WM, Tilg H, Van Hul M, Cani PD (2022). Gut microbiome and health: mechanistic insights. Gut.

[20] van der Meulen TA, Harmsen H, Bootsma H, Spijkervet F, Kroese F, Vissink A (2016). The microbiome-systemic diseases connection. Oral Dis.

[21] Sorbara MT, Pamer EG (2022). Microbiome-based therapeutics. Nat Rev Microbiol.

[22] O’Toole PW, Marchesi JR, Hill C (2017). Next-generation probiotics: the spectrum from probiotics to live biotherapeutics. Nat Microbiol.

[23] Cani PD, de Vos WM (2017). Next-generation beneficial microbes: the case of *Akkermansia muciniphila*. Front Microbiol.

[24] Douillard FP, de Vos WM (2019). Biotechnology of health-promoting bacteria. Biotechnol Adv.

[25] Eckburg PB, Bik EM, Bernstein CN, Purdom E, Dethlefsen L, Sargent M (2005). Diversity of the human intestinal microbial flora. Science.

[26] Rajilić-Stojanović M, Smidt H, de Vos WM (2007). Diversity of the human gastrointestinal tract microbiota revisited. Environ Microbiol.

[27] Turroni F, Peano C, Pass DA, Foroni E, Severgnini M, Claesson MJ (2012). Diversity of bifidobacteria within the infant gut microbiota. PLoS ONE.

[28] Penders J, Thijs C, Vink C, Stelma FF, Snijders B, Kummeling I (2006). Factors influencing the composition of the intestinal microbiota in early infancy. Pediatrics.

[29] Korpela K, de Vos WM (2018). Early life colonization of the human gut: microbes matter everywhere. Curr Opin Microbiol.

[30] Lebeaux RM, Madan JC, Nguyen QP, Coker MO, Dade EF, Moroishi Y (2022). Impact of antibiotics on off-target infant gut microbiota and resistance genes in cohort studies. Pediatr Res.

[31] Korpela K, Salonen A, Virta LJ, Kekkonen RA, Forslund K, Bork P (2016). Intestinal microbiome is related to lifetime antibiotic use in Finnish pre-school children. Nat Commun.

[32] Hopkins MJ, Macfarlane GT, Furrie E, Fite A, Macfarlane S (2005). Characterisation of intestinal bacteria in infant stools using real-time PCR and northern hybridisation analyses. FEMS Microbiol Ecol.

[33] Long SS (2001). Infant botulism. Pediatr Infect Dis J.

[34] Arnon SS (1980). Infant Botulism. Ann Rev Med.

[35] Huhtanen CM (1979). Bile acid inhibition of *Clostridium botulinum*. Appl Environ Microbiol.

[36] Lam TI, Tam CC, Stanker LH, Cheng LW. Probiotic microorganisms inhibit epithelial cell internalization of botulinum neurotoxin serotype A. Toxins (Basel). 2016;8(12). 10.3390/toxins8120377 10.3390/toxins8120377 PMC5198571 27999281

[37] Francisco AM, Arnon SS (2007). Clinical mimics of infant botulism. Pediatrics.

[38] Harris RA, Anniballi F, Austin JW. Adult intestinal toxemia botulism. Toxins (Basel). 2020;12(2). 10.3390/toxins12020081 10.3390/toxins12020081 PMC7076759 31991691

[39] Mills DC, Arnon SS (1987). The large intestine as the site of *Clostridium botulinum* colonization in human infant botulism. J Infect Dis.

[40] Rosow LK, Strober JB (2015). Infant botulism: review and clinical update. Pediatr Neurol.

[41] Freedman M, Armstrong RM, Killian JM, Boland D (1986). Botulism in a patient with jejunoileal bypass. Ann Neurol.

[42] Fenicia L, Anniballi F, Aureli P (2007). Intestinal toxemia botulism in Italy, 1984–2005. Eur J Clin Microbiol Infect Dis.

[43] Griffin PM, Hatheway CL, Rosenbaum RB, Sokolow R (1997). Endogenous antibody production to botulinum toxin in an adult with intestinal colonization botulism and underlying Crohn’s disease. J Infect Dis.

[44] Arnon SS, Midura TF, Clay SA, Wood RM, Chin J (1977). Infant botulism. Epidemiological, clinical, and laboratory aspects. JAMA.

[45] Arnon SS, Midura TF, Damus K, Thompson B, Wood RM, Chin J (1979). Honey and other environmental risk factors for infant botulism. J Pediatr.

[46] Nevas M, Hielm S, Lindström M, Horn H, Koivulehto K, Korkeala H. High prevalence of *Clostridium botulinum* types A and B in honey samples detected by polymerase chain reaction. Int J Food Microbiol. 2002;72(1–2):45–52. 10.1016/s0168-1605(01)00615-8 10.1016/s0168-1605(01)00615-8 11843412

[47] Spika JS, Shaffer N, Hargrett-Bean N, Collin S, MacDonald KL, Blake PA (1989). Risk factors for infant botulism in the United States. Am J Dis Child.

[48] Nevas M, Lindström M, Virtanen A, Hielm S, Kuusi M, Arnon SS (2005). Infant botulism acquired from household dust presenting as sudden infant death syndrome. J Clin Microbiol.

[49] Panditrao MV, Dabritz HA, Kazerouni NN, Damus KH, Meissinger JK, Arnon SS (2020). Seven-year case-control study in California of risk factors for infant botulism. J Pediatr.

[50] Lindström M, Korkeala H (2006). Laboratory diagnostics of botulism. Clin Microbiol Rev.

[51] Scheithauer TPM, Rampanelli E, Nieuwdorp M, Vallance BA, Verchere CB, van Raalte DH, et al. Gut microbiota as a trigger for metabolic inflammation in obesity and type 2 diabetes. Front Immunol. 2020;11. 10.3389/fimmu.2020.571731 10.3389/fimmu.2020.571731 PMC7596417 33178196

[52] Gurung M, Li Z, You H, Rodrigues R, Jump DB, Morgun A, et al. Role of gut microbiota in type 2 diabetes pathophysiology. eBioMedicine. 2020;51. 10.1016/j.ebiom.2019.11.051 10.1016/j.ebiom.2019.11.051 PMC6948163 31901868

[53] Qiu P, Ishimoto T, Fu L, Zhang J, Zhang Z, Liu Y. The gut microbiota in inflammatory bowel disease. Front Cell Infect Microbiol. 2022;12. 10.3389/fcimb.2022.733992 10.3389/fcimb.2022.733992 PMC8902753 35273921

[54] Pascal V, Pozuelo M, Borruel N, Casellas F, Campos D, Santiago A (2017). A microbial signature for Crohn’s disease. Gut.

[55] Rebersek M (2021). Gut microbiome and its role in colorectal cancer. BMC Cancer.

[56] Flemer B, Lynch DB, Brown JMR, Jeffery IB, Ryan FJ, Claesson MJ (2017). Tumour-associated and non-tumour-associated microbiota in colorectal cancer. Gut.

[57] Shirey TB, Dykes JK, Lúquez C, Maslanka SE, Raphael BH (2015). Characterizing the fecal microbiota of infants with botulism. Microbiome.

[58] Derman Y, Korkeala H, Salo E, LÖNnqvist T, Saxen H, Lindström M (2014). Infant botulism with prolonged faecal excretion of botulinum neurotoxin and *Clostridium botulinum* for 7 months. Epidemiol Infect.

[59] Dabritz HA, Payne JR, Khouri JM. Duration of fecal excretion of *Clostridium botulinum* and botulinum neurotoxin in children recovering from infant botulism. J Pediatr. 2023;113396. 10.1016/j.jpeds.2023.113396 10.1016/j.jpeds.2023.113396 37004956

[60] Douillard FP, Derman Y, Woudstra C, Selby K, Mäklin T, Dorner MB (2022). Genomic and phenotypic characterization of *Clostridium botulinum* isolates from an infant botulism case suggests adaptation signatures to the gut. mBio.

[61] Jian C, Luukkonen P, Yki-Järvinen H, Salonen A, Korpela K (2020). Quantitative PCR provides a simple and accessible method for quantitative microbiota profiling. PLoS ONE.

[62] Raju SC, Lagström S, Ellonen P, de Vos WM, Eriksson JG, Weiderpass E (2018). Reproducibility and repeatability of six high-throughput 16S rDNA sequencing protocols for microbiota profiling. J Microbiol Methods.

[63] Luukkonen PK, Sädevirta S, Zhou Y, Kayser B, Ali A, Ahonen L (2018). Saturated fat is more metabolically harmful for the human liver than unsaturated fat or simple sugars. Diabetes Care.

[64] Martin M. Cutadapt removes adapter sequences from high-throughput sequencing reads. 2011. 2011;17(1):3; 10.14806/ej.17.1.200

[65] Callahan BJ, McMurdie PJ, Rosen MJ, Han AW, Johnson AJ, Holmes SP (2016). DADA2: high-resolution sample inference from Illumina amplicon data. Nat Methods.

[66] Quast C, Pruesse E, Yilmaz P, Gerken J, Schweer T, Yarza P (2013). The SILVA ribosomal RNA gene database project: improved data processing and web-based tools. Nucleic Acids Res.

[67] McLaren MR, Callahan BJ. Silva 138.1 prokaryotic SSU taxonomic training data formatted for DADA2. 2021.

[68] Johnson M, Zaretskaya I, Raytselis Y, Merezhuk Y, McGinnis S, Madden TL. NCBI BLAST: a better web interface. Nucleic Acids Res. 2008;W5–9. 10.1093/nar/gkn201. 36(Web Server issue). 10.1093/nar/gkn201 PMC2447716 18440982

[69] Oksanen J, Blanchet FG, Kindt R, Legendre P, Minchin PR, O’Hara RB et al. vegan: community ecology package. In.; 2012.

[70] Parker RI, Vannest KJ, Davis JL, Sauber SB (2011). Combining nonoverlap and trend for single-case research: Tau-U. Behav Ther.

[71] Lahti L, Salojärvi J, Salonen A, Scheffer M, de Vos WM (2014). Tipping elements in the human intestinal ecosystem. Nat Comm.

[72] Arrieta MC, Stiemsma LT, Amenyogbe N, Brown EM, Finlay B (2014). The intestinal microbiome in early life: health and disease. Front Immunol.

[73] Laursen MF, Bahl MI, Michaelsen KF, Licht TR (2017). First Foods and Gut microbes. Front Microbiol.

[74] Matharu D, Ponsero AJ, Dikareva E, Korpela K, Kolho KL, de Vos WM (2022). Bacteroides abundance drives birth mode dependent infant gut microbiota developmental trajectories. Front Microbiol.

[75] Xiao L, Wang J, Zheng J, Li X, Zhao F (2021). Deterministic transition of enterotypes shapes the infant gut microbiome at an early age. Genome Biol.

[76] Belzer C, de Vos WM (2012). Microbes inside—from diversity to function: the case of Akkermansia. ISME J.

[77] Fujihashi K, Staats HF, Kozaki S, Pascual DW (2007). Mucosal vaccine development for botulinum intoxication. Expert Rev Vaccines.

[78] Kobayashi R, Kohda T, Kataoka K, Ihara H, Kozaki S, Pascual DW (2005). A novel neurotoxoid vaccine prevents mucosal botulism. J Immunol.

[79] Cooksley CM, Davis IJ, Winzer K, Chan WC, Peck MW, Minton NP (2010). Regulation of neurotoxin production and sporulation by a putative *agrBD* signaling system in proteolytic *Clostridium botulinum*. Appl Environ Microbiol.

[80] Schechter R, Peterson B, McGee J, Idowu O, Bradley J (1999). *Clostridium difficile* colitis associated with infant botulism: near-fatal case analogous to Hirschsprung’s enterocolitis. Clin Infect Dis.

[81] Fenicia L, Da Dalt L, Anniballi F, Franciosa G, Zanconato S, Aureli P (2002). A case of infant botulism due to neurotoxigenic *Clostridium butyricum* type E associated with *Clostridium difficile* colitis. Eur J Clin Microbiol Infect Dis.

[82] Domingo RM, Haller JS, Gruenthal M (2008). Infant botulism: two recent cases and litterature review. J Child Neurol.

[83] Reuter G (2001). The *Lactobacillus* and *Bifidobacterium* microflora of the human intestine: composition and succession. Curr Issues Intest Microbiol.

[84] Picard C, Fioramonti J, Francois A, Robinson T, Neant F, Matuchansky C (2005). Review article: bifidobacteria as probiotic agents -- physiological effects and clinical benefits. Aliment Pharmacol Ther.

[85] Favier CF, Vaughan EE, De Vos WM, Akkermans AD (2002). Molecular monitoring of succession of bacterial communities in human neonates. Appl Environ Microbiol.

[86] Gueimonde M, Debor L, Tölkkö S, Jokisalo E, Salminen S (2007). Quantitative assessment of faecal bifidobacterial populations by real-time PCR using lanthanide probes. J Appl Microbiol.

[87] Sullivan NM, Mills DC, Riemann HP, Arnon SS (1988). Inhibition of growth of *Clostridium botulinum* by intestinal microflora isolated from healthy infants. Microb Ecol Health Dis.

