# Supplementary figures and images for: Case report: Aberrant fecal microbiota composition of an infant diagnosed with prolonged intestinal botulism
Source: Gut Pathog. 2024 Apr 5;16:20. doi: 10.1186/s13099-024-00614-y (PMC10996148; doi:10.1186/s13099-024-00614-y)

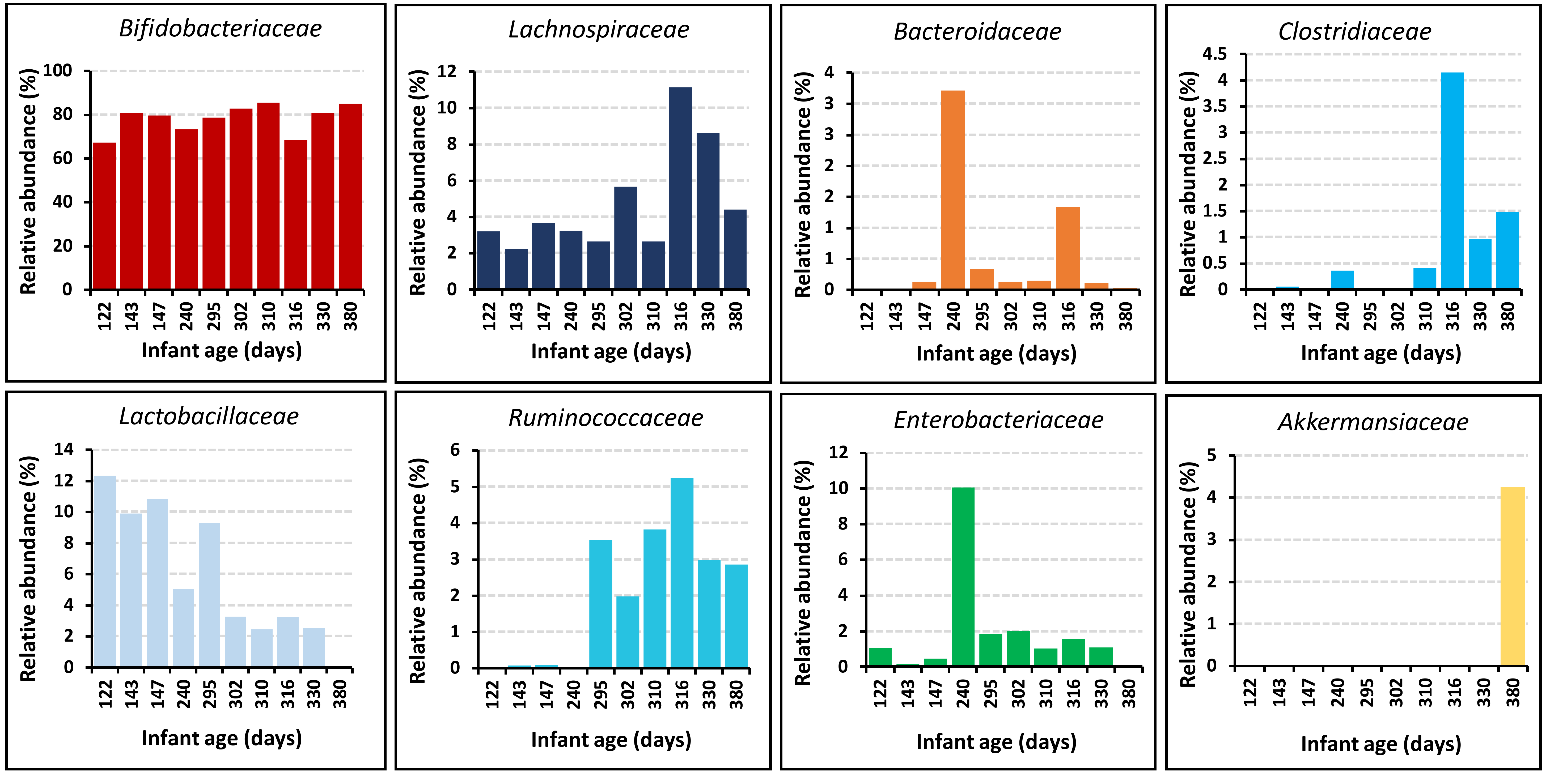

Supplement: Supplementary file 1 — Supplementary Figure S1: Relative abundance of relevant bacterial families over time [file 13099_2024_614_MOESM1_ESM.png]
