# Supplementary material for: Case report: Aberrant fecal microbiota composition of an infant diagnosed with prolonged intestinal botulism
Source: Gut Pathog. 2024 Apr 5;16:20. doi: 10.1186/s13099-024-00614-y (PMC10996148; doi:10.1186/s13099-024-00614-y)
Supplement: Supplementary file 2 — Supplementary Table S1: 16S rRNA gene sequencing statistics of all fecal samples analyzed in the present study. Only the first three sample were collected when the infant was at the hospital [file 13099_2024_614_MOESM2_ESM.docx]

**Table S1.** 16S rRNA gene sequencing statistics of all fecal samples analyzed in the present study. Only the first three sample were collected when the infant was at the hospital. Subsequent samples were collected at home.

| **Sample #** | **Infant age (days)** | **BioSample accession number** | **SRA number** |  | **Total reads** |
| --- | --- | --- | --- | --- | --- |
| 1  2  3  4  5  6  7  8  9  10 | 122  143  147  240  295  302  310  316  330  380 | SAMN32638839  SAMN32638840  SAMN32638841  SAMN32638842  SAMN32638843  SAMN32638844  SAMN32638845  SAMN32638846  SAMN32638847  SAMN32638848 | SRX18975747  SRX18975748  SRX18975749  SRX18975750  SRX18975751  SRX18975752  SRX18975753  SRX18975754  SRX18975755  SRX18975756 |  | 30050  29294  31566  37991  29590  65164  77064  54678  86311  37178 |
